# The effects of dietary calcium and phosphorus level, and feed form during rearing on growth performance, bone traits and egg production in brown egg-type pullets from 0 to 32 weeks of age

**DOI:** 10.1016/j.psj.2021.101130

**Published:** 2021-03-16

**Authors:** M.A. Dijkslag, R.P. Kwakkel, E. Martin-Chaves, C. Alfonso-Carrillo, C. Walvoort, A. Navarro-Villa

**Affiliations:** ⁎ Animal Nutrition Group, Wageningen University, NL-6700 AH, Wageningen, the Netherlands; † Trouw Nutrition R&D, Poultry Research Centre, El Viso de San Juán, Toledo, 45950, Spain; ‡ Nutrition and Innovation Centre, ForFarmers N.V., NL-7240 AB, Lochem, the Netherlands

**Keywords:** egg-type pullet, phosphorus, calcium, feed form, egg production

## Abstract

In a 3 × 2 factorial arrangement, effects of feed form (crumbles (**CWS**), mash (**MWS**), both with inclusion of 3% finely ground wheat straw, or crumbles with inclusion of 3% oat hulls (**COH**)), and dietary Ca and P (high and low Ca-P) from 0 to 16 wk of age were studied on growth performance, bone characteristics, and gizzard development of egg-type pullets. The cross-over effect of feeding strategy during rearing on laying performance and egg shell quality was studied from 19 to 32 wk of age. From 0 to 16 wk, ADG, ADFI, and feed conversion ratio (**FCR**) were improved with CWS and COH compared to MWS, but ADG and FCR were improved with MWS compared to CWS and COH from 11 to 16 wk. Uniformity of BW till 11 wk, and tibia breaking strength at 6 and 16 wk were higher with CWS and COH compared to MWS. Tibia ash content at 11 wk and relative empty proventriculus + gizzard weight (EPG) were lower with CWS and COH compared to MWS, also relative EPG at 11 and 16 wk was higher with COH compared to CWS. At 25 wk BW was lower with MWS compared to CWS and COH, but BW was equal for all treatments at 32 wk. The FCR for egg production was improved with COH compared to MWS. Egg shell parameters were not affected by feed form during rearing. Low Ca-P decreased BW uniformity at 6 wk, relative keel bone weight and ash content at 11 wk, tibia ash content at 11 and 16 wk, increased relative EPG at 6 wk, and improved egg shell quality at 32 wk of age. It was concluded that feeding CWS and COH compared to MWS increased growth performance, but had no clear cross-over effect on egg production. Low dietary Ca-P led to a lower bone mineralization during rearing, nevertheless improved egg shell quality at 32 wk.

## INTRODUCTION

Calcium (**Ca**) and Phosphorus (**P**) are essential elements in poultry nutrition. On one hand, Ca is essential for bone and egg shell formation, blood clotting, muscle contraction and transmission of nerve impulses. Also, Ca is an important co-factor for many enzymes and hormones (Li et al., 2016). On the other hand, P is required for normal muscle growth and egg formation, is an important component of nucleic acids, the genetic code, and phospholipids, and is also a co-factor of many enzyme systems. In addition, P plays a vital role in maintaining osmotic and acid base balance, energy metabolism, amino acid metabolism and protein synthesis (Li et al., 2016). During skeletal growth and bone remodeling, Ca and P are required for the formation of hydroxyapatite and other mineral-phase components. The rate at which mineralization occurs is dependent, in part, on the availability of P and Ca (Berndt et al., 2005). An oversupply of dietary Ca to growing chickens has been shown to decrease growth rate in both broilers and egg-type pullets (Miller and Sunde, 1975; Shafey, 1993; Akter et al., 2016; Gautier et al., 2017). An oversupply of dietary P is costly to the poultry industry and leads to an of excessive P discharge to the environment via manure application to soils (Jing et al., 2018). These authors showed that it is possible to reduce P in egg-type pullet diets without impairing growth or affecting bone characteristics. Also our research group found in a previous study no negative effects on growth and bone characteristics of low P diets in egg-type pullets fed from 16-27 wk of age, but low dietary P resulted in small negative effects on egg weight and egg mass production during the start of lay (Dijkslag et al., 2019). Bradbury et al. (2014) showed significant interactions of Ca and P levels in broiler diets. At low Ca and high P, as well as high Ca and low P diets, birds had reduced ADFI, ADG, poorer feed conversion ratio (**FCR**) and lower tibia ash. This proves that the dietary Ca to P ratio is an important factor in growing bird nutrition. It has been shown in broilers that chickens are able to adapt to early dietary changes in P and Ca through improvement of digestive efficiency in a later phase, and the extent of the compensation in terms of growth performance and bone mineralization depends on the P and Ca levels in the subsequent diets (Rousseau et al., 2016). Punna and Roland (1999) showed that low dietary P levels during the rearing phase of egg-type pullets did not impair egg production performance, when fed sufficient P during the laying period.

The use of fiber in poultry diets has gained attention in recent years. Moderate amounts of fiber (2 to 4%) in the diet of egg-type pullets improves bird performance, although the effect of different fiber sources varies (Guzmán et al., 2015a). Dietary fiber may increase energy efficiency, ADG, ADFI and gizzard development (Guzmán et al., 2015a,b). Positive effects of increasing dietary fiber have also been found in broilers. Several authors reported improved ADG, FCR and increased gizzard weights (Gonzáles-Alvarado et al., 2010; Jiménez-Moreno et al., 2010; Sacranie et al., 2012, 2013). Sacranie et al. (2012) also reported a lower gizzard pH in broilers fed a diet with 15% oat hulls (**OH**). Structural components, such as OH, improve not only gizzard musculature tone, grinding activity and digesta retention time in the proventriculus and gizzard, but also decrease digesta pH in the gizzard thereby improving nutrient utilization (Svihus, 2011, 2014) and phytate P solubility (Nahashon et al., 1994).

Feed form offered to egg-type pullets has a strong effect on bird performance. Several trials showed that, compared to mash, pelleted (Deaton et al., 1988; Frikha et al., 2009a,b) or crumbled feed (Guzmán et al., 2015a; Saldaña et al., 2015a,b) resulted in an increased ADG due to a higher ADFI. Additionally, feeding pelleted diets reduced the relative weight of the gastrointestinal tract (**GIT**) and increased gizzard pH, due to the decreased dietary particle size, as occurred when the diets were pelleted (Frikha et al., 2009a; Saldaña et al., 2015a).

Limited data are available on the dietary requirement of Ca and P in egg-type pullets during rearing and the cross-over effects on egg production at later ages. Since feed form and dietary fiber, both source and structure, may affect feed intake and digestive efficiency, it is likely that these factors could affect net mineral intake and metabolism as well. Therefore, the objective of this study was to evaluate high and low combined dietary Ca and P (**Ca-P**) levels, fed as mash or crumbles, with or without coarse OH, on growth and bone development during the rearing period from 0-16 wk of age and their cross-over effect on egg production from 19-32 wk of age.

## MATERIALS AND METHODS

The protocol for the experiment conformed to the standards for animal experiments and was approved by the Trouw Nutrition animal care committee and followed recommendations of the Junta de Castilla-La Mancha (Spain) Animal Welfare department as stated in the royal decree RD 53/2013 (Boletín Oficial del Estado, 2013).

### Birds and Husbandry

A total number of 1,380 day old female Bovans Brown egg-type pullets were obtained from a commercial hatchery (Société Française de Production Avicole, Saint-Marcellin, France). Birds were housed in a semi-commercial rearing facility (Granja Agas S.A., Cuenca, Spain). The birds had intact beaks and were assigned to 6 different treatment groups, which were replicated (n=10) in adjacent cages, each of them allocating a total of 23 pullets on day 0. A total of 10 experimental blocks, with 6 adjacent cages per block, were pre-stablished based on the location in the barn. The dimensions of each cage were 100 × 65 × 40 cm (length × width × height) and contained a feeding trough and 2 drinking nipples per cage. Feed and water were provided *ad libitum*. Pullets were kept on a 18 h/d light program for the first wk of life. Then, the light period was decreased by 1 h/wk from the second wk until reaching 12 h/d in wk 6 and this was continued till the end of the rearing period. Light intensity was 40 lux in the first wk of life and was decreased to 20 lux (wk 2), 10 lux (wk 3), and 6 lux from wk 4 onwards. Temperature was set at 35˚C on arrival and was gradually decreased to 21˚C at day 23 until the end of the rearing period. Birds were vaccinated against main diseases (Infectious Bronchitis, Mycoplasma, Gumboro, Salmonella, New Castle Disease, Egg Drop Syndrome) according to accepted commercial practices.

At 16 wk of age, birds were transferred to an experimental layer house facility (Trouw Nutrition Poultry Research Centre, Casarrubios del Monte, Spain) with 2 separate, but identical rooms. Each room contained a 2 level battery with 24 enriched cages with a capacity for 20 birds per cage. The animals from each cage in the rearing facility remained together as experimental unit when transferred to the layer facility. The assignment of cages in the layer facility followed a complete randomized experimental block design based on room, battery level and location in the room. Due to capacity limits, the number of replicates was decreased from 10 to 8 and selection was based on lowest mortality rate during rearing. The dimensions of each cage were 241 × 62.5 × 45 cm (length × width × height) and contained a scratch pad, perches, a laying nest and 4 drinking nipples. After arrival at the layer facility, light was increased with 1/wk from 12 h/d till 15 h/d day at 19 wk of age. Light intensity was initially set at 20 lux, but was decreased till 8 lux to avoid feather pecking behavior, as birds were nervous and showed signs of aggression. This was maintained till the end of the experiment. Rooms were climate controlled and temperature was set at 20˚C.

### Dietary Treatments

**Figure 1:**
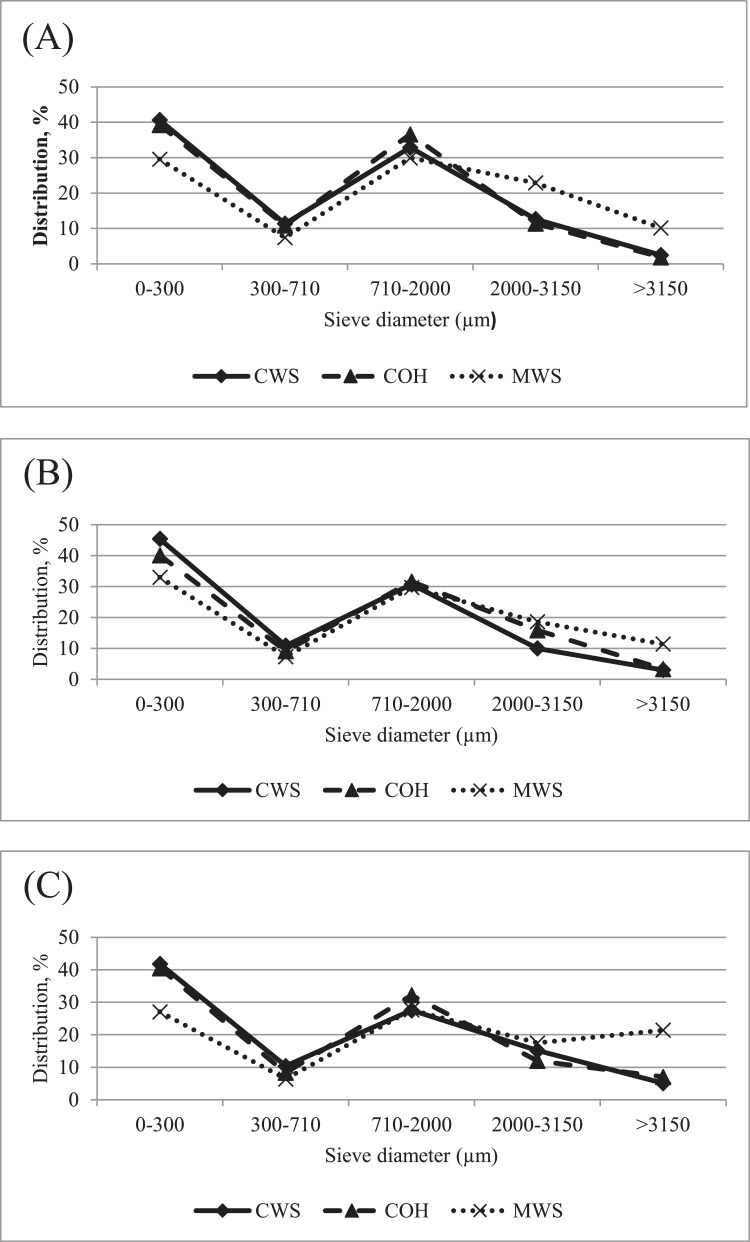
Particle size distribution (determined with wet sieving) of the rearing diets from 0 to 6 wk (A), 6 to 11 wk (B) and 11 to 16 wk of age (C) diets. Abbreviations: CWS, crumbles; COH, crumbles with 3% coarse oat hulls; MWS, mash.

Dietary treatments were arranged in a 3 × 2 factorial arrangement with 2 factors: feed form (mash with inclusion of 3% finely ground wheat straw (**MWS**) vs. crumbles with inclusion of 3% finely ground wheat straw (**CWS**) vs. crumbles with inclusion of 3% unground OH as fiber source (**COH**)), and Ca-P content (high vs. low levels), which are presented in Table 1. The experimental diets were fed during the rearing period (0 to 16 wk of age). All experimental rearing diets were produced as basal mixture per phase from raw materials of the same batch. For each treatment, a blend of the corresponding test ingredients was premixed and incorporated to the basal mixture. Depending on the targeted feed form, diets were pelleted to 3 mm pellet diameter and crushed to produce crumbles or, alternatively only mixed to produce mash. Particle size distributions were determined by wet sieve analyses (American National Standards Institute/American Society of Agricultural Engineers, 2008) and particle size distributions per feed form are presented in Figure 1. The COH diets were produced by replacing 3% finely ground wheat straw (**WS**) by unground OH, as ground cereal straw has been reported not to have a positive effect on growth performance and gizzard development (Guzmán et al., 2015a,b) and was therefore chosen as control ingredient. The high dietary Ca-P levels were considered being adequate for growth and development of rearing egg-type pullets (approximately 90% of CVB (2016) recommendation). The feeding schedule consisted of 3 phases during the rearing period (0 to 6 wk, 6 to 11 wk, and 11 to 16 wk of age). The ingredient compositions of the experimental rearing diets with the calculated and analyzed compositions are presented in Table 2. During the laying period all birds received a commercial layer diet (Nanta, Griñón, Spain) with adequate dietary Ca and P, which was fed as crumbles. The ingredient compositions of the experimental layer diets with the calculated and analyzed compositions are presented in Table 3. Samples of experimental diets were analyzed according to AOAC International 18^th^ edition (2005), for DM content by oven-drying (934.01), total ash (942.05), nitrogen by combustion (990.03) using a LECO analyzer and ether extract (960.39). Samples were also analyzed for starch by the α-amylase glucosidase method (996.11), crude fiber by sequential extraction with diluted acid and alkali (962.09), and Ca and P by flame absorption spectrophotometry (965.17).

**Table 1:** Overview dietary treatments in the rearing phase from 0 to 16 wk of age.

Label treatment	Feed form	Fiber source (3%)^3^	Ca (g/kg)^1^	rP (g/kg)^2^
1. MWS - High Ca-P	Mash	WS	7.5-6.7-7.3	3.6-3.2-3.0
2. MWS - Low Ca-P	Mash	WS	5.8-4.7-5.0	3.2-2.6-2.4
3. CWS - High Ca-P	Crumbles	WS	7.5-6.7-7.3	3.6-3.2-3.0
4. CWS - Low Ca-P	Crumbles	WS	5.8-4.7-5.0	3.2-2.6-2.4
5. COH - High Ca-P	Crumbles	OH	7.5-6.7-7.3	3.6-3.2-3.0
6. COH - Low Ca-P	Crumbles	OH	5.8-4.7-5.0	3.2-2.6-2.4

1 Dietary Ca content from respectively 0 to 6, 6 to 11, and 11 to 16 wk of age.

2 Dietary retainable P content from respectively 0 to 6, 6 to 11, and 11 to 16 wk of age.

3 WS: fine wheat straw; OH: coarse oat hulls.

**Table 2:** Composition, calculated and determined analysis (g/kg, as-fed) of experimental diets fed to pullets from 0 to 16 wk of age.

	0-6 wk				6-11 wk				11-16 wk			
Treatment^1^	1&3	2&4	5	6	1&3	2&4	5	6	1&3	2&4	5	6
Ingredient												
Corn	300.0	300.0	300.0	300.0	300.0	300.0	300.0	300.0	300.0	300.0	300.0	300.0
Wheat	351.8	351.8	351.8	351.8	404.0	404.0	404.0	404.0	423.8	423.8	423.8	423.8
Soybean meal	262.7	262.7	262.7	262.7	217.9	217.9	217.9	217.9	199.3	199.3	199.3	199.3
Soya oil	22.3	22.3	22.3	22.3	17.1	17.1	17.1	17.1	12.0	12.0	12.0	12.0
Sodiumbicarbonate	2.8	2.8	2.8	2.8	2.9	2.9	2.9	2.9	2.8	2.6	2.6	2.6
Methionine Hydroxy Analog	2.7	2.7	2.7	2.7	2.2	2.2	2.2	2.2	1.3	1.3	1.3	1.3
Sodiumchloride	2.1	2.1	2.1	2.1	1.9	1.9	1.9	1.9	1.9	1.9	1.9	1.9
Lysine (Biolys 70)	1.0	1.0	1.0	1.0	1.4	1.4	1.4	1.4	0.2	0.2	0.2	0.2
L-Threonine 98%	0.6	0.6	0.6	0.6	0.5	0.5	0.5	0.5	0.1	0.1	0.1	0.1
Calciumcarbonate	11.3	7.9	11.3	7.8	10.3	6.5	10.3	6.5	11.5	7.1	11.5	7.1
Monocalciumphosphate	6.7	4.8	6.7	4.8	4.9	1.6	4.9	1.6	4.1	0.9	4.1	0.9
Sepiolite 15/30		5.3		5.3	0.5	7.7	0.6	7.8	2.0	9.3	2.0	9.3
Premix^3^	5.0	5.0	5.0	5.0	5.0	5.0	5.0	5.0	5.0	5.0	5.0	5.0
Premix phytase^3^	1.0	1.0	1.0	1.0	1.0	1.0	1.0	1.0	1.0	1.0	1.0	1.0
Wheat straw fine	30.0	30.0			30.0	30.0			30.0	30.0		
Oat hulls coarse			30.0	30.0			30.0	30.0			30.0	30.0
Calculated analysis												
AMEn, kcal/kg^4^	2,698	2,698	2,698	2,698	2,697	2,697	2,697	2,697	2,699	2,699	2,699	2,699
CP	185.3	185.3	185.3	185.3	165.3	165.3	165.0	165.0	157.0	157.0	156.7	156.7
Ca	7.5	5.8	7.5	5.8	6.7	4.7	6.7	4.7	7.3	5.0	7.3	5.0
P	5.1	4.7	5.1	4.7	4.6	3.8	4.6	3.9	4.3	3.6	4.3	3.6
rP^2^	3.6	3.2	3.6	3.2	3.2	2.6	3.2	2.6	3.0	2.4	3.0	2.4
Determined analysis^5^												
Ca	8.7/7.8	7.2/7.3	8.0	6.2	11.9/9.3	8.4/6.7	8.9	6.7	8.6/7.7	5.9/5.7	7.8	5.7
P	5.1/4.9	4.8/4.8	5.2	4.8	4.9/4.6	3.8/3.6	4.5	3.6	4.0/3.8	3.3/3.3	4.0	3.2

1 Treatment 1: mash, high Ca and P; Treatment 2: mash, low Ca and P; Treatment 3: crumbles, high Ca and P; Treatment 4: crumbles, low Ca and P; Treatment 5: crumbles with 3% oat hulls, high Ca and P; Treatment 6: crumbles with 3% oat hulls, low Ca and P.

2 rP = retainable phosphorus (CVB, 2016).

3 Provided per kilogram of complete diet: vitamin A, 10,000 IU; vitamin D3, 2,500 IU; vitamin E, 50 IU; vitamin K3, 2.0 mg; vitamin B1, 2.0 mg; vitamin B2, 2.0 mg; vitamin B6, 4.0 mg; vitamin B12, 0.03 mg; niacinamide, 40 mg; D-pantothenic acid, 10 mg; folic acid, 1.0 mg; biotin, 0.15 mg; choline, 260 mg; iron, 67.7 mg (as FeSO_4_·7H_2_O); copper, 15 mg (as CuSO_4_·5H_2_O); manganese, 90 mg (as MnSO_4_·H_2_O); zinc, 80 mg (as ZnO); iodine, 1.0 mg (as CaI); selenium, 0.25 mg (as Na_2_SeO_3_·5H_2_O); Phytase, 500 FTU, (supplied by Trouw Nutrition Spain).

4 AMEn is based on calculation methods from CVB (2016) for chickens.

5 For treatment 1-4, two figures are displayed. The first was determined in mash, the second in crumbles.

**Table 3:** Composition, calculated and determined analysis (g/kg) of the experimental diets during the laying period (17 to 32 wk of age).

Age period diets were fed	17-26 wk	26-32 wk
Ingredient		
Barley	50.0	50.0
Corn	350.0	577.5
Wheat	110.8	
Rapeseed meal	100.0	80.0
Soybean meal	124.4	134.1
Hominy feed (Corn)	100.0	
Wheat bran	12.8	
Soybean oil		7.3
Corn DDGS	50.0	50.0
Calciumcarbonate	91.7	90.2
Monocalciumphosphate	1.9	2.0
Sodiumchloride	2.7	2.8
Methionine Hydroxy Analog	1.2	1.4
Lysine (Biolys 70)	0.6	0.5
L-Tryptophan		0.1
Premix Canthaxanthin^1^	0.8	0.8
Choline chloride (75%)	0.2	0.4
Premix^2^	3.0	3.0
Calculated analysis		
AMEn, kcal/kg^3^	2650	2800
CP	170.0	160.0
Ca	38.8	37.0
P	4.2	4.0
rP^4^	3.3	3.1
Determined analysis		
CP	169.9	165.5
Ca	38.0	32.4
P	3.9	3.6

1 Provided per kilogram of complete diet: 3.4 mg canthaxanthin, (supplied by Trouw Nutrition Spain).

2 Provided per kilogram of complete diet: vitamin A, 7,500 IU; vitamin D3, 1,500 IU; vitamin E, 6 IU; vitamin K3, 2.0 mg; vitamin B1, 2.0 mg; vitamin B2, 3.0 mg; vitamin B6, 3.0 mg; vitamin B12, 0.03 mg; niacinamide, 20 mg; D-pantothenic acid, 6.5 mg; folic acid, 0.5 mg; biotin, 0.1 mg; choline, 295 mg; iron, 40 mg (as FeSO_4_·7H_2_O); copper, 12 mg (as CuSO_4_·5H_2_O); manganese, 90 mg (as MnSO_4_·H_2_O); zinc, 60 mg (as ZnO); iodine, 1.0 mg (as CaI); selenium, 0.20 mg (as Na_2_SeO_3_·5H_2_O); Phytase, 600 FTU, (supplied by Trouw Nutrition Spain).

3 AMEn is based on calculation methods from CVB (2016) for laying hens.

### Observations

#### Performance Parameters

The key parameters under evaluation of the current study were ADG, ADFI, FCR, egg production and flock uniformity. In order to evaluate these, individual BW was determined at the end of each feeding phase. Flock uniformity was determined by calculating the percentage of birds within the range mean ±10% average BW per experimental unit. Therefore, BW, ADG, ADFI, FCR and flock uniformity per experimental unit were calculated at the end of wk 6, 11 and 16. The FCR was calculated as the ratio between ADFI and ADG for the rearing period (0 to 16 wk), and the ratio between ADFI and average daily egg mass production for the laying period (19 to 32 wk).

#### Necropsies

At the end of each feeding phase (6, 11, 16, and 32 wk of age), one pullet per experimental unit (within the ±95% average BW in that unit) was euthanized by cervical dislocation. Right tibias were removed for analysis of breaking strength and ash content, as well as the keel bone that was removed for ash content determination. Proventriculus and gizzards were removed and weighed to determine the empty proventriculus + gizzard weight (**EPG**) relative to BW, as an indication of the degree of development of these organs.

#### Bone and Egg Parameters

Tibia weight, breaking strength, and ash content were recorded at 6, 11, 16 and 32 wk of age. Keel bone weight and ash content were recorded at 6, 11, and 16 wk of age. To determine ash contents, tibias and keel bones were weighed, placed in pre-weighed crucibles and dried for 18 h at 103˚C. After dry matter weight was recorded, bones were ashed in a muffle oven at 550˚C for 720 minutes. Crucibles were cooled down to room temperature in silica desiccators and weighed to record ash content. At 22, 25, and 32 wk of age, 4 intact eggs per pen were randomly taken to evaluate eggshell quality (breaking strength, shell weight, shell thickness). Tibia and egg shell breaking strength, and egg shell thickness were recorded by using a TA.XT plus100C texture analyzer (Stable Micro Systems, Godalming, UK). Shell weight per unit surface area (**SWUSA**) was calculated as egg shell weight (mg) divided by egg shell surface area, where surface area was calculated by the equation (Phirinyane et al., 2011):

$$\text{SA}=3.9782\times \left(W^{0.7056}\right)$$

where SA is the surface sarea in cm^2^ and W is egg weight in g.

### Statistical Analysis

Raw data were analyzed for outliers (mean ± 2.5 SD). Significant outliers were not included in the mean results and statistical analysis. Pen was the experimental unit for performance data, while pullet was the experimental unit for bone and EPG data. Measurements were evaluated by analysis of variance using GenStat (14^th^ edition, VSNI, Hemel Hempstead, UK) statistical software according the following general model:

$$Y_{\text{ijk}}=\mu +\text{Feed}\text{for}m_{i}+\text{Ca}-Plevel_{i}+Feedform_{i}\times Ca-Plevel_{j}+Block_{k}+e_{ijk}$$

where Y_ijk_ is the measured response, μ is the overall mean effect, Feed form_i_ is the fixed feed form effect (i = MWS, CWS, or COH) and Ca-P level_j_ is the fixed Ca-P level (j = high or low). Row (rearing phase) or room (laying phase) number was added as factor into the model only when its effect was significant (*P* < 0.05). All interactions between Feed form_i_ and Ca-P level_j_ were included and e_ijk_ is the error associated with the i^th^ feed form and the j^th^ Ca-P level. The P-value of the statistical model is given per response parameter. The null hypothesis was that there was no treatment effect on the response parameter. Treatment means were compared by least significant difference (LSD) after significant effects were confirmed by ANOVA. Values with *P* ≤ 0.05 were considered to be statistically significant.

## RESULTS

### Rearing Period

#### Growth Performance and BW Uniformity

Mortality was 2.2% during the rearing period and was not related to any of the treatments (data not shown). The effects of dietary treatments during the rearing period (0 to 16 wk of age) on growth performance and BW uniformity are presented in Table 4.

**Table 4:** Growth performance and BW uniformity parameters of egg-type pullets fed diets as crumbles (CWS), crumbles with 3% coarse oat hulls (COH), or mash (MWS) feed form, and high or low Ca and P level from 0 to 16 wk of age.

	0 to 6 wk			6 to 11 wk			11 to 16 wk			0 to 16 wk			BW Uniformity^1^		
Effect	ADFI (g/d)	ADG (g/d)	Feed conversion ratio (g/g)	ADFI (g/d)	ADG (g/d)	Feed conversion ratio (g/g)	ADFI (g/d)	ADG (g/d)	Feed conversion ratio (g/g)	ADFI (g/d)	ADG (g/d)	Feed conversion ratio (g/g)	6 wk of age (%)	11 wk of age (%)	16 wk of age (%)
Feed form															
CWS	29.4^a^	11.9^a^	2.48^b^	67.6^a^	18.7^a^	3.63^b^	81.8	11.3^b^	7.23^a^	55.9^a^	13.9^a^	4.04^b^	80.3^a^	83.8^a^	87.8
COH	29.5^a^	12.0^a^	2.46^b^	68.2^a^	18.4^a^	3.73^a^	81.2	11.3^b^	7.22^a^	55.5^a^	13.7^a^	4.08^a,b^	78.8^a^	81.8^a^	84.9
MWS	27.9^b^	10.7^b^	2.62^a^	62.8^b^	17.2^b^	3.68^b^	79.6	11.6^a^	6.87^b^	53.6^b^	13.1^b^	4.14^a^	71.0^b^	71.5^b^	87.3
SEM (n = 20)	0.20	0.07	0.017	0.50	0.13	0.022	0.63	0.07	0.049	0.31	0.04	0.021	1.88	2.17	2.09
Ca and P level															
High	29.0	11.6	2.51	66.6	18.1	3.71	81.1	11.4	7.10	55.2	13.6	4.08	78.9^a^	79.3	88.6
Low	28.9	11.4	2.53	65.8	18.1	3.65	80.6	11.4	7.11	54.8	13.5	4.09	74.4^b^	78.7	84.7
SEM (n = 30)	0.16	0.06	0.014	0.41	0.11	0.018	0.51	0.06	0.040	0.25	0.04	0.017	1.53	1.77	1.71
Feed form × Ca and P level															
CWS – High	29.3	12.0	2.46	67.7	18.5	3.69	81.7	11.3	7.21	55.9	13.9	4.03	82.7	83.6	88.3
CWS – Low	29.4	11.8	2.50	67.6	18.8	3.58	81.8	11.3	7.24	55.9	13.8	4.05	77.8	83.9	87.4
COH – High	29.8	12.1	2.47	69.0	18.3	3.76	81.3	11.3	7.17	55.6	13.7	4.09	80.5	83.1	88.6
COH – Low	29.2	11.9	2.46	67.4	18.5	3.69	81.1	11.2	7.27	55.4	13.7	4.07	77.1	80.4	81.2
MWS – High	27.9	10.7	2.62	63.2	17.4	3.66	80.3	11.6	6.92	54.1	13.1	4.16	73.7	71.1	89.1
MWS – Low	27.9	10.7	2.62	62.4	17.1	3.70	79.0	11.6	6.82	53.1	13.0	4.12	68.3	71.8	85.4
SEM (n = 10)	0.28	0.10	0.023	0.70	0.19	0.031	0.89	0.10	0.070	0.44	0.06	0.029	2.66	3.07	2.95
Source of variation	*P*-values														
Feed form	<.001	<.001	<.001	<.001	<.001	0.022	0.058	0.001	<.001	<.001	<.001	0.020	0.002	<.001	0.579
Ca and P level	0.611	0.195	0.453	0.160	0.779	0.053	0.540	0.383	0.904	0.270	0.105	0.536	0.042	0.826	0.107
Feed form × Ca and P level	0.444	0.627	0.521	0.571	0.169	0.057	0.712	0.630	0.366	0.466	0.647	0.597	0.930	0.835	0.553

^a-b^Means within a column and within a source without a common superscript differ significantly (*P* < 0.05).

1 Evaluated as the percentage of birds within ±10% of the average BW.

In general, only feed form significantly affected growth performance throughout the rearing period. From 0 to 6 wk of age, ADFI was respectively 1.5 g/d and 1.6 g/d higher in the CWS and COH fed pullets compared to MWS (*P* < 0.001). During this period, ADG was respectively 1.2 g/d and 1.3 higher in the CWS and COH fed pullets compared to MWS (*P* < 0.001), which resulted in an improved FCR of respectively 0.141 g/g and 0.156 g/g in the CWS and COH fed pullets compared to MWS (*P* < 0.001). Similar effects were noticed from 6 to 11 wk of age for ADFI and ADG. During this period, ADFI was respectively 4.8 g/d and 5.4 g/d higher, and ADG was respectively 1.5 g/d and 1.2 g/d higher in the CWS and COH fed pullets compared to MWS (*P* < 0.001). However, FCR was improved with respectively 0.094 g/g and 0.049 g/g in the CWS and MWS fed pullets, compared to COH (*P* = 0.022). Feed form did not significantly affect ADFI from 11 to 16 wk of age, however ADG was 0.3 g/d higher in MWS fed pullets compared to CWS and COH (*P* = 0.001). As a consequence of this, FCR was improved (*P* < 0.001) with MWS compared to CWS and COH with respectively 0.358 g/g and 0.351 g/g. During the whole rearing period (0 to 16 wk), ADFI was respectively 2.3 g/d and 1.9 g/d higher in the CWS and COH fed pullets compared to MWS (*P* < 0.001), and ADG was respectively 0.8 g/d and 0.6 g/d higher in the CWS and COH fed pullets compared to MWS (*P* < 0.001). This resulted in a lower FCR of 0.095 g/g in the CWS fed pullets compared to MWS (*P* = 0.020), while COH was intermediate. MWS fed pullets showed a lower BW uniformity compared to CWS (9.3%) and COH (7.8%) at 6 wk of age (*P* = 0.002). Also at 11 wk of age, BW uniformity was lower with MWS, compared to CWS (12.3%) and COH (10.3%) fed pullets (*P* < 0.001). At 16 wk of age, BW uniformity was not affected by feed form.

At 6 wk of age BW uniformity was 4.5% improved in the high compared to the low dietary Ca-P level fed pullets. At 11 and 16 wk of age, BW uniformity was not affected by dietary Ca-P level. None of the other parameters in any of the age intervals (ADFI, ADG, FCR) were affected by dietary Ca-P level.

#### Bone Parameters

The effects of dietary treatments on keel bone parameters during the rearing period are presented in Table 5. At 11 wk of age dietary Ca-P affected keel bone characteristics. The low Ca-P level resulted in a 0.019% higher relative keel bone weight (*P* = 0.025) and keel bone ash was 2.0% higher in pullets fed high compared to low dietary Ca-P (*P* = 0.006). There was a feed form × Ca-P level interaction on keel bone ash content (% of DM) at 16 wk of age (*P* = 0.030). Within birds fed the COH diet, keel bone ash was 2.9% higher when high dietary Ca-P was given compared to low, where keel bone ash was unaffected by dietary Ca-P level when fed the CWS or MWS diets.

**Table 5:** Keel bone parameters at 6, 11, and 16 wk of age of egg-type pullets fed diets as crumbles (CWS), crumbles with 3% coarse oat hulls (COH), or mash (MWS) feed form, and high or low Ca and P level.

	6 wk		16 wk		16 wk	
Effect	Relative weight (% of BW)	Ash (% of DM)	Relative weight (% of BW)	Ash (% of DM)	Relative weight (% of BW)	Ash (% of DM)
Feed form						
CWS	0.26	41.0	0.29	46.7	0.32	47.7
COH	0.26	40.8	0.30	46.1	0.33	47.5
MWS	0.25	40.5	0.29	47.1	0.35	47.8
SEM (n = 20)	0.006	0.66	0.007	0.61	0.010	0.49
Ca and P level						
High	0.25	40.6	0.28^b^	47.6^a^	0.33	48.0
Low	0.26	40.9	0.30^a^	45.6^b^	0.33	47.3
SEM (n = 30)	0.005	0.54	0.006	0.50	0.009	0.40
Feed form × Ca and P level						
CWS – High	0.26	40.5	0.27	48.0	0.32	47.4^a,b^
CWS – Low	0.25	41.4	0.30	45.4	0.32	48.1^a^
COH – High	0.26	40.4	0.30	47.2	0.32	48.9^a^
COH – Low	0.26	41.2	0.30	44.9	0.33	46.0^b^
MWS – High	0.26	41.0	0.27	47.6	0.35	47.8^ab^
MWS – Low	0.25	40.0	0.31	46.5	0.34	47.8^ab^
SEM (n = 10)	0.009	0.94	0.010	0.86	0.015	0.69
Source of variation	*P*-values					
Feed form	0.740	0.899	0.363	0.505	0.195	0.880
Ca and P level	0.371	0.737	0.025	0.006	0.892	0.207
Feed form × Ca and P level	0.618	0.521	0.088	0.661	0.866	0.030

^a-b^Means within a column and within a source without a common superscript differ significantly (*P* < 0.05).

The effects of dietary treatments on tibia parameters during the rearing period are presented in Table 6. At 6 wk of age, tibia breaking strength was lower (P = 0.011) in the MWS compared to CWS (1.52 kg) and COH (1.47 kg) fed pullets. Feed form affected tibia ash content at 11 wk of age. Tibia ash content (% of DM) was 1.5% lower in both CWS and COH compared to MWS fed pullets (*P* = 0.008). At 16 wk of age, tibia breaking strength was 2.50 kg higher in COH compared to MWS fed pullets (*P* = 0.039). Tibia ash content was 1.4% higher in pullets fed high compared to low dietary Ca-P at 11 wk of age (*P* = 0.003). Tibia ash content was 0.7% higher (*P* = 0.045) when fed the high compared to low dietary Ca-P level at 16 wk of age.

**Table 6:** Tibia parameters at 6, 11, and 16 wk of age of egg-type pullets fed diets as crumbles (CWS), crumbles with 3% coarse oat hulls (COH), or mash (MWS) feed form, and high or low Ca and P level.

	6 wk				16 wk				16 wk			
Effect	Relative weight (% of BW)	Ash (% of DM)	Breaking strength (kg)	Compression (mm)	Relative weight (% of BW)	Ash (% of DM)	Breaking strength (kg)	Compression (mm)	Relative weight (% of BW)	Ash (% of DM)	Breaking strength (kg)	Compression (mm)
Feed form												
CWS	0.67	41.6	11.39^a^	2.33	0.69	36.8^b^	25.48	2.77	0.73	32.7	23.17^a,b^	2.45
COH	0.67	41.0	11.34^a^	2.35	0.67	36.8^b^	23.37	2.61	0.72	32.5	23.78^a^	2.47
MWS	0.64	40.8	9.87^b^	2.25	0.68	38.3^a^	23.27	2.73	0.75	32.8	21.28^b^	2.54
SEM (n = 20)	0.010	0.53	0.385	0.051	0.012	0.39	1.102	0.098	0.010	0.30	0.698	0.131
Ca and P level												
High	0.66	41.2	11.23	2.33	0.67	38.0^a^	24.46	2.69	0.73	33.0^a^	23.37	2.51
Low	0.66	41.1	10.50	2.29	0.69	36.6^b^	23.62	2.72	0.73	32.3^b^	22.11	2.47
SEM (n = 30)	0.008	0.43	0.314	0.041	0.010	0.31	0.900	0.080	0.008	0.24	0.570	0.107
Feed form × Ca and P level												
CWS – High	0.66	41.4	11.14	2.27	0.67	37.7	26.13	2.76	0.72	32.7	23.01	2.35
CWS – Low	0.68	41.8	11.64	2.39	0.72	35.8	24.83	2.78	0.74	32.6	23.33	2.55
COH – High	0.67	41.2	12.42	2.40	0.68	37.7	24.95	2.64	0.73	33.4	25.27	2.45
COH – Low	0.67	40.8	10.26	2.30	0.66	35.8	21.79	2.58	0.72	31.6	22.28	2.49
MWS – High	0.66	40.9	10.13	2.32	0.67	38.5	22.29	2.76	0.75	32.9	21.83	2.71
MWS – Low	0.63	40.7	9.61	2.18	0.70	38.1	24.25	2.71	0.74	32.7	20.73	2.36
SEM (n = 10)	0.014	0.75	0.544	0.071	0.017	0.55	1.558	0.138	0.014	0.42	0.987	0.186
Source of variation	*P*-values											
Feed form	0.110	0.572	0.011	0.322	0.261	0.008	0.287	0.476	0.312	0.813	0.039	0.895
Ca and P level	0.656	0.938	0.109	0.447	0.147	0.003	0.515	0.775	0.925	0.045	0.127	0.805
Feed form × Ca and P level	0.351	0.851	0.058	0.153	0.118	0.300	0.262	0.957	0.599	0.080	0.254	0.323

^a-b^Means within a column and within a source without a common superscript differ significantly (*P* < 0.05).

#### Relative Empty Proventriculus + Gizzard Weight

The effects of dietary treatments on relative EPG (% of BW) are presented in Table 7. Pullets fed MWS had a higher relative EPG (3.78%) compared to CWS (3.29%) and COH (3.49%) (*P* < 0.001) at 6 wk of age. Relative EPG was higher in MWS (3.13%) compared to COH (2.74%) fed pullets, with CWS (2.57%) having a lower relative EPG than with both MWS and COH fed pullets (*P* <0.001) at 11 wk of age. Relative EPG was higher in MWS (2.63%) compared to COH (2.25%) fed pullets, with CWS (2.09%) having a lower relative EPG compared to both MWS and COH fed pullets (*P* < 0.001) at 16 wk of age. Relative EPG was higher in low (3.62%) compared to high (3.41%) dietary Ca-P fed pullets (*P* = 0.014) at 6 wk of age.

**Table 7:** Relative empty proventriculus + gizzard weight (as % of BW) of egg-type pullets fed diets as crumbles (CWS), crumbles with 3% coarse oat hulls (COH), or mash (MWS) feed form, and high or low Ca and P level at 6, 11, and 16 wk of age.

	Relative empty gizzard + proventriculus weight (% of BW)		
Effect	6 wk	11 wk	16 wk
Feed form			
CWS	3.29^b^	2.57^c^	2.09^c^
COH	3.49^b^	2.74^b^	2.25^b^
MWS	3.78^a^	3.13^a^	2.63^a^
SEM (n = 20)	0.072	0.055	0.058
Ca and P level			
High	3.41^b^	2.79	2.28
Low	3.62^a^	2.85	2.37
SEM (n = 30)	0.059	0.045	0.047
Feed form × Ca and P level			
CWS – High	3.16	2.56	2.08
CWS – Low	3.42	2.59	2.09
COH – High	3.36	2.67	2.21
COH – Low	3.61	2.82	2.30
MWS – High	3.72	3.14	2.54
MWS – Low	3.84	3.13	2.72
SEM (n = 10)	0.102	0.077	0.082
Source of variation	*P*-values		
Feed form	<.001	<.001	<.001
Ca and P level	0.014	0.381	0.166
Feed form × Ca and P level	0.713	0.563	0.591

^a-b^Means within a column and within a source without a common superscript differ significantly (*P* < 0.05).

### Laying Period

#### Egg Production

Mortality was 0.9% during the laying period and was not related to any of the treatments (data not shown). The effects of dietary treatments during the rearing period on the egg production during the laying period (19 to 32 wk of age) are presented in Table 8. Feed form during the rearing period affected point of lay 50%. When fed CWS or COH, pullets reached 50% laying rate respectively 1.0 and 1.1 d earlier compared to the MWS fed birds (*P* = 0.018). Feed form during the rearing period affected FCR in the laying period. When fed COH, FCR was 0.037 g/g lower, compared to CWS fed birds (*P* = 0.033). At 25 wk of age, BW was 39 and 33 g lower (*P* = 0.017) of the MWS fed birds compared to the CWS and COH fed birds respectively.

**Table 8:** Egg production and BW parameters of laying hens from 19 to 32 wk of age, fed diets during rearing (0 to 16 wk of age) as crumbles (CWS), crumbles with 3% coarse oat hulls (COH), or mash (MWS) feed form, and high or low Ca and P levels.

Effect^1^	Rate of lay (%)	Point of lay 50% (d)	ADFI (g/d)	Egg weight (g)	Egg mass production (g/d)	FCR^2^ (g/g)	BW wk 25 (g)	BW wk 32 (g)	BW uniformity^3^ wk 25 (%)	BW uniformity^3^ wk 32 (%)
Feed form										
CWS	94.1	130.3^b^	114.0	55,9	52.6	2.17^a^	1821^a^	1898	83.6	72.7
COH	94.7	130.2^b^	112.9	56.0	53.0	2.13^b^	1815^a^	1895	83.7	73.4
MWS	93.5	131.3^a^	113.9	56.3	53.1	2.15^ab^	1782^b^	1898	77.9	79.0
SEM (n = 16)	0.57	0.28	0.56	0.16	0.28	0.010	9.7	9.9	1.96	2.87
Ca and P level										
High	94.2	130.6	113.2	56.0	52.8	2.14	1803	1899	82.5	77.8
Low	94.0	130.6	114.0	56.1	53.0	2.15	1810	1895	81.0	72.2
SEM (n = 24)	0.47	0.23	0.46	0.13	0.22	0.008	8.0	8.0	1.60	2.34
Feed form × Ca and P level										
CWS – High	94.3	129.9	113.9	56.0	52.8	2.16	1818	1905	83.2	77.4
CWS – Low	93.8	130.8	114.0	55.9	52.4	2.18	1824	1891	84.1	68.0
COH – High	94.5	130.3	112.0	55.6	52.6	2.13	1804	1894	86.9	74.5
COH – Low	94.9	130.1	113.8	56.3	53.4	2.13	1826	1896	80.6	72.2
MWS – High	93.8	131.7	113.6	56.3	53.0	2.14	1786	1900	77.5	81.7
MWS – Low	93.2	131.0	114.2	56.2	53.1	2.15	1779	1898	78.4	76.4
SEM (n = 8)	0.81	0.40	0.80	0.23	0.39	0.014	13.8	13.9	2.77	4.05
Source of variation	*P*-values									
Feed form	0.376	0.018	0.369	0.338	0.428	0.033	0.017	0.960	0.071	0.241
Ca and P level	0.701	0.970	0.207	0.410	0.569	0.447	0.528	0.688	0.507	0.098
Feed form × Ca and P level	0.803	0.151	0.524	0.107	0.251	0.737	0.563	0.844	0.335	0.678

^a-b^Means within a column and within a source without a common superscript differ significantly (*P* < 0.05).

1 Treatments were applied during rearing (0 to 16 wk of age). All diets were similar in composition and fed as crumbles after 16 wk of age.

2 Feed conversion ratio for egg production (ADFI/Egg mass production).

3 Evaluated as the percentage of birds within ±10% of the average BW.

#### Tibia Characteristics

The effects of the dietary treatments during the rearing period on the tibia characteristics at 32 wk of age are presented in Table 9. Feed form during the rearing period affected tibia breaking strength. When fed MWS, breaking strength was 5.10 g higher compared to COH, with CWS as intermediate (*P* = 0.018). There was a feed form × Ca-P level interaction on tibia compression (*P* = 0.023). When fed COH, tibia compression was 0.36 mm less when fed the low compared to high Ca-P level during rearing. Compression was not affected by Ca-P level when fed MWS or CWS.

**Table 9:** Tibia parameters of laying hens at 32 wk of age, fed diets during rearing (0 to 16 wk of age) as crumbles (CWS), crumbles with 3% coarse oat hulls (COH), or mash (MWS) feed form, and high or low Ca and P level.

	Tibia			
Effect^1^	Relative weight (% of BW)	Ash (% of DM)	Breaking strength (kg)	Compression (mm)
Feed form				
CWS	0.62	41.0	23.36^ab^	1.92
COH	0.63	40.2	20.71^b^	1.97
MWS	0.62	42.2	25.80^a^	1.96
SEM (n = 16)	0.010	0.73	1.194	0.078
Ca and P level				
High	0.63	41.3	24.05	2.01
Low	0.63	40.9	22.54	1.89
SEM (n = 24)	0.009	0.60	0.975	0.063
Feed form × Ca and P level				
CWS – High	0.61	40.4	24.17	2.04^ab^
CWS – Low	0.64	41.6	22.55	1.81^b^
COH – High	0.63	40.1	21.86	2.15^a^
COH – Low	0.63	40.3	19.56	1.79^b^
MWS – High	0.63	43.5	26.11	1.83^ab^
MWS – Low	0.61	41.0	25.50	2.08^ab^
SEM (n = 8)	0.015	1.04	1.689	0.110
Source of variation	*P*-values			
Feed form	0.749	0.169	0.018	0.926
Ca and P level	0.929	0.642	0.282	0.200
Feed form × Ca and P level	0.112	0.208	0.882	0.023

^a-b^Means within a column and within a source without a common superscript differ significantly (*P* < 0.05).

1 Treatments were applied during rearing (0 to 16 wk of age). All diets were similar in composition and fed as crumbles after 16 wk of age.

#### Egg Characteristics

The effects of the dietary treatments during the rearing period on egg shell quality and percentage of abnormal eggs (broken, dirty, shell less, small, deformed, and double yolked), are presented in Table 10. The Ca-P level during rearing affected egg shell quality at 32 wk of age. Egg shell breaking strength was 300 g higher (*P* = 0.021), egg shell thickness was 0.011 mm thicker (*P* < 0.001), and SWUSA was 1.9 mg/cm^2^ higher (*P* = 0.009) with the low compared to the high dietary Ca-P level during the rearing period.

**Table 10:** Egg shell quality parameters at 22, 25, and 32 wk of age, and percentage of abnormal eggs of eggs from 19 to 32 wk of age from laying hens fed diets during rearing (0 to 16 wk of age) as crumbles (CWS), crumbles with 3% coarse oat hulls (COH), or mash (MWS) feed form, and high or low Ca and P level.

	22 wk			25 wk			32 wk			19 – 32 wk			
Effect^1^	Egg shell breaking strength (g)	Egg shell thick-ness (mm)	SWUSA (mg/cm^2^)	Egg shell breaking strength (g)	Egg shell thick-ness (mm)	SWUSA (mg/cm^2^)	Egg shell breaking strength (g)	Egg shell thick-ness (mm)	SWUSA (mg/cm^2^)	Broken eggs (%)	Dirty eggs (%)	Shell less eggs (%)	Other abnormal eggs (%)
Feed form													
CWS	5850	0.398	84.6	6000	0.402	84.3	5990	0.392	85.9	0.73	3.05	0.57	0.28
COH	6020	0.404	85.6	5860	0.407	85.4	6140	0.398	86.7	0.85	2.56	0.59	0.36
MWS	5850	0.395	83.9	6010	0.403	84.7	6150	0.399	87.0	0.82	2.67	0.59	0.35
SEM (n = 16)	99.1	0.0034	0.62	112.2	0.0035	0.71	123.8	0.0027	0.59	0.109	0.285	0.062	0.046
Ca and P level													
High	6020	0.401	84.9	6000	0.406	85.2	5940^b^	0.391^b^	85.6^b^	0.79	2.76	0.55	0.35
Low	5800	0.397	84.4	5920	0.402	84.4	6240^a^	0.402^a^	87.5^a^	0.81	2.76	0.62	0.31
SEM (n = 24)	80.9	0.0028	0.50	91.6	0.0029	0.58	101.1	0.0022	0.49	0.089	0.233	0.051	0.038
Feed form × Ca and P													
CWS – High	5910	0.400	84.3	6060	0.404	84.2	5980	0.383	84.5	0.60	3.02	0.55	0.30
CWS – Low	5800	0.396	85.0	5930	0.400	84.3	6000	0.402	87.3	0.86	3.07	0.58	0.26
COH – High	6090	0.407	85.5	5910	0.407	85.5	5850	0.391	85.6	0.82	2.53	0.50	0.38
COH – Low	5960	0.402	85.6	5810	0.407	85.2	6420	0.405	87.8	0.88	2.59	0.68	0.33
MWS – High	6060	0.398	85.1	6020	0.407	85.8	6010	0.397	86.7	0.94	2.72	0.60	0.37
MWS – Low	5650	0.392	82.7	6010	0.399	83.6	6290	0.400	87.4	0.69	2.61	0.58	0.33
SEM (n = 8)	140.1	0.0048	0.87	158.7	0.0050	1.00	175.1	0.0039	0.84	0.154	0.403	0.088	0.065
Source of variation	*P*-values												
Feed form	0.386	0.138	0.175	0.568	0.590	0.551	0.159	0.193	0.406	0.744	0.457	0.319	0.407
Ca and P level	0.063	0.228	0.483	0.529	0.330	0.343	0.021	<.001	0.009	0.848	0.998	0.912	0.410
Feed form × Ca and P level	0.488	0.962	0.182	0.925	0.659	0.447	0.175	0.136	0.434	0.253	0.970	0.589	0.988

Abbreviations: SA, surface area; SWUSA, shell weight per unit surface area.

^a-b^Means within a column and within a source without a common superscript differ significantly (*P* < 0.05).

1 Treatments were applied during rearing (0 to 16 wk of age). All diets were similar in composition and fed as crumbles after 16 wk of age.

## DISCUSSION

### Feed Form

#### Productive Performance 0-16 wk

Pullets fed MWS had a lower ADFI compared to feeding CWS and COH (Table 4). These findings are in agreement with Saldaña et al. (2015a,b) and Frikha et al. (2009a,b), who found a lower ADFI with mash compared to pelleted feed, fed to brown egg-type pullets from 0 to 17 wk of age. Guzmán et al. (2015a) found only a lower ADFI with mash compared to crumbles in the first wk of life in brown egg-type pullets, but from 3 to 5 wk of age ADFI was higher with mash diets. These authors hypothesized that beak trimming, which was performed immediately post hatch, might have reduced feed intake early in life in mash fed birds. This explanation is however not applicable to the current study as birds were not beak trimmed. The effect of feed form on ADFI of the current trial is in contrast to the findings of Bozkurt et al. (2019), who found a higher ADFI when feeding mash compared to crumbles in white egg-type pullets till 16 wk of age. Also Gous and Morris (2001) found a higher ADFI with mash compared to pelleted diets fed to brown egg-type pullets. Deaton et al. (1988) found no difference in ADFI between pellets and mash with egg-type pullets from 12 to 20 wk of age. Although the effect of feed form on ADFI of egg-type pullets is not found consistent, the majority of the mentioned studies, including the present experiment, showed an increased ADFI with pelleted diets, which confirms the hypothesis that poultry require more time and energy for compacting feed particles when the diets are presented as mash than when presented as crumbles (Saldaña et al., 2015a). Pelleting also increases bulk density and facilitates feed intake (Frikha et al., 2009b).

The current trial showed an increased ADG when pullets were fed CWS or COH, compared to MWS from 0 to 16 wk (Table 4). These findings are in agreement with several authors who found an increased ADG in egg-type pullets fed crumbles compared to mash diets (Guzmán et al., 2015a; Saldaña et al., 2015a,b; Bozkurt et al., 2019) or pellets compared to mash diets (Deaton et al., 1988; Gous and Morris, 2001; Frikha et al., 2009a,b). The increase of ADG is probably due to the increased ADFI with the crumbled diets, but also the modifying effects of pelleting the feed on dietary starch and protein structure that increases energy and protein digestibility might have contributed (Saldaña et al., 2015a).

Although ADFI was increased when fed CWS and COH from 0 to 16 wk of age, FCR was improved compared to MWS (Table 4). This was due to the increased ADG with the CWS and COH fed pullets. These findings are in agreement with data from several authors (Gous and Morris, 2001; Guzmán et al., 2015a; Saldaña et al., 2015a,b; Bozkurt et al., 2019) who also found an improved FCR with pelletized compared to mash diets in egg-type pullets. Frikha et al. (2009a,b), however, did not find a difference in FCR between crumbles and mash fed pullets till 45 d of age. Bozkurt et al. (2019) found the improved FCR with a lower ADFI. The effect was explained by a higher digestibility coefficient for ether extract, an increased amylase and lipase activity, and an increased ileal absorption surface area, when crumbles were fed, compared to mash. Ege et al. (2019) also found an increased amylase activity and ileal surface area in laying hens fed crumbles, which indicate an increased nutrient utilization. The argument of the increased ileal surface area by both authors is indeed an indication for an increased nutrient absorption capacity, but it is worth noting that the method used only calculates the surface area of an average villus and does not take other factors (e.g. intestinal length, number of villi per intestinal surface area and feed passage rate) into account. The latter makes it unfeasible to prove that the absorption capacity of the gut was actually increased. On the other hand, the increased enzymatic activity found by these authors might as well explain the improved FCR in the present study. The effect of OH on technical performance is well described in research with broiler chickens, where improved performance is reported when OH were included in the diet (Gonzáles-Alvardo et al., 2010; Jiménez-Moreno et al., 2010; Sacranie et al., 2012). These findings are, however, in contrast with the findings of the present study, as the FCR of the COH fed pullets was intermediate between CWS and MWS and numerically higher compared to CWS from 0 to 16 wk. The lack of improvement is hard to explain as broiler research reported an increased nutrient retention when OH were included, which explained an improved FCR. In the present study, OH were exchanged with finely ground WS as feed ingredient and both are fibrous ingredients. Cereal straw has been reported not to have a positive effect on growth performance (Guzmán et al., 2015a,b) and was therefore chosen as control ingredient in the present study. Despite the fact that OH stimulated proventriculus + gizzard development better than WS in the current study, it did not have a positive effect on growth performance and FCR. Feed form (crumbles compared to mash) seemed to have a larger impact on these parameters.

#### Age Effect of Feed Form

**Figure 2:**
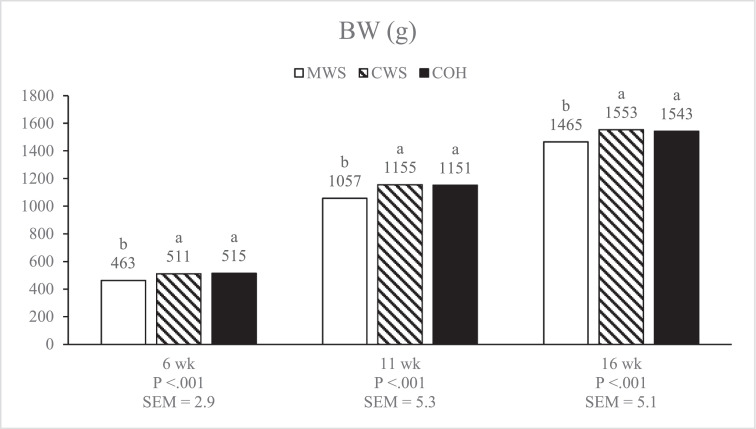
BW of egg-type pullets fed diets as mash (MWS), crumbles (CWS), or crumbles with 3% coarse oat hulls (COH) feed form from 0 to 16 wk of age.

The present study showed that the effect of feed form on ADG changed over time as birds aged. The ADG was only increased with CWS and COH fed pullets till 11 wk of age, and FCR was improved till 6 wk of age. From 6 to 11 wk of age FCR was improved in both MWS and CWS compared to COH, whereas in the final period (11 to 16 wk), MWS fed pullets had a higher ADG and improved FCR compared to CWS and COH (Table 4). This means that BW of MWS fed pullets was respectively 10% and 9% lower at 6 and 11 wk of age compared to CWS fed pullets, but this difference was reduced to 6% at 16 wk of age (Figure 2). Bozkurt et al. (2019) reported a lower ADG and worse FCR in white egg-type pullets till 8 wk of age when fed mash, compared to crumbles, but no difference for both parameters from 8 to 16 wk. Comparable results are reported by Frikha et al. (2009a,b) in brown egg type pullets, where young birds showed lower growth performance when fed mash compared to pellets, but across time as birds aged no effect of feed form was observed. Other studies showed an increased ADG and improved FCR in crumbles fed egg type pullets throughout the rearing period, but the difference was more pronounced at younger age (Saldaña et al., 2015a,b). Although the age effect of feed form on growth performance in egg type pullets was more pronounced in the present study than in comparable experiments, we may conclude that pullets at older age grow faster and have a better FCR on mash compared to young birds. The effect might be related to the difference in proventriculus + gizzard development. At 6 wk of age the relative EPG of CWS fed birds was 87% of the relative EPG of MWS, at 11 wk it was 82%, and at 16 wk of age it was reduced to 79% (Table 7). Pullets fed MWS had better proventriculus + gizzard development and the difference compared to CWS increased with age. Also, MWS fed pullets might therefore have increased their digestive capacity more with age, thereby potentially increasing their voluntary feed intake capacity compared to crumbles fed birds.

#### BW Uniformity

The current trial showed an improved BW uniformity till 11 wk of age of CWS and COH compared to MWS, but at 16 wk of age feed form did not affect BW uniformity anymore (Table 4). These findings are quite similar to Saldaña et al. (2015b), who found a tendency for a better BW uniformity when crumbles instead of mash was fed to egg-type pullets at 5wk of age, but the difference disappeared when birds grew older. In contrast to that, Saldaña et al. (2015a) found in another experiment a better BW uniformity with crumbles compared to mash diet at 10 and 17 wk, but no significant difference at 5 wk of age. Bozkurt et al. (2019) found no effect of feed form on BW uniformity in egg-type pullets. The effect of feed form on BW uniformity is not consistent, but the effect in the present study might be due to the previously mentioned hypothesis that MWS fed pullets might have increased their digestive capacity more with age compared to crumbles fed birds and were therefore better capable of improving this parameter.

#### Bone Parameters

Tibia ash content was found to be higher in MWS compared to CWS and COH fed pullets at 11 wk of age (Table 6). As to the authors’ knowledge, no data is available on the effect of feed form on bone mineralization in egg-type pullets. However, Kilburn and Edwards (2001) found in their first experiment a higher bone ash content in mash compared to pellets fed broiler chickens, but in their second experiment feed form did not affect bone ash content. Their first experiment also showed an interaction of feed form and corn grinding method. The effect was more pronounced when corn was coarsely ground, so diet particle size seems to affect bone mineralization as well. The effect of feed form on bone mineralization remains unclear as in the present study the effect was only significant at 11 wk of age, while at 6 wk tibia ash content was numerically lower in MWS compared to CWS and COH, and at 16 wk there was no difference at all. Tibia breaking strength appeared to be higher when pullets were fed CWS and COH compared to MWS at 6 and 16 wk of age. The MWS fed birds had a lower growth rate and the relative tibia weight (% of BW) was not affected by feed form, so MWS fed birds had smaller tibias. As bone strength is proportional to its mass (Rath et al., 2000), the difference in breaking strength was most likely due to the lower tibia mass of the MWS fed birds.

#### Proventriculus + Gizzard Development

Feed form was found to have a clear effect on relative EPG. At all ages measured, CWS fed pullets had the lowest, and MWS fed pullets the highest relative EPG, while COH fed pullets were intermediate (Table 7). These findings are similar to the results reported by several authors who compared pelleted and mash diets in egg-type pullets (Frikha et al., 2009a,b; Saldaña et al., 2015a,b; Bozkurt et al., 2019) and laying hens (Ege et al., 2019). The main driver for poultry gizzard development seems to be diet particle size and birds do not fully develop their gastro intestinal tract when highly processed pelleted feeds are used (Zaefarian et al., 2016). Also data from the present study confirmed this, as relative EPG was the highest with MWS and these diets had the highest content of coarse (>2000 μm) particles (Figure 1). Pullets seem to adapt relatively quick to changes in feed form (Svihus, 2011; Saldaña et al., 2015b). This was also reported in internal research work in which a higher relative gizzard weight was found in egg-type pullets at 22 and 28 wk of age, when coarse instead of fine mash diets were fed from wk 16 onwards (Dijkslag et al., 2019). At 11 and 16 wk of age relative EPG was higher with COH compared to CWS fed pullets. The addition of structural fibrous materials in poultry diets has been proven an efficient method to stimulate gizzard development in broiler chickens (Gonzáles-Alvardo et al., 2010; Jiménez-Moreno et al., 2010; Sacranie et al., 2012, 2013; Jiménez-Moreno et al., 2019), and this effect seems to be present in egg-type pullets as well.

#### Laying Period: Cross-Over Effects From the Rearing Period

When pullets were fed MWS, BW was lower during rearing (Figure 2). Nevertheless, BW at 16 wk of age with MWS was at target according to the Bovans Brown management guide (www.bovans.com), but BW of CWS and COH fed pullets was approximately 5% above target. At 25 wk of age, BW of the MWS fed birds during rearing was still lower compared to CWS and COH, but at 32 wk of age, BW of all feed form treatments were comparable (Table 8). Pullets fed MWS during rearing showed compensatory growth during the laying period, when all treatments were offered the same crumbled layer diet. Compensatory growth in egg-type pullets has been reported by Johnson et al. (1984) and appeared also in the present trial.

Relative tibia weight at 32 wk of age was not affected by feed form during rearing, but tibia breaking strength appeared to be higher at this age when pullets were fed MWS compared to COH (Table 9). This is probably related to the compensatory growth of the MWS fed birds, as bone strength is proportional to its mass (Rath et al., 2000), but also tibia ash content seemed to be related to breaking strength. Pullets fed COH during rearing had numerically the lowest tibia ash content and lowest breaking strength as MWS fed pullets showed the highest levels for both parameters.

Feed form during the rearing period affected point of lay 50%. When fed MWS, point of lay 50% was delayed with approximately 1 d compared to CWS and COH (Table 8), but did not affect egg production. Birds fed MWS had a lower BW at 16 and 25 wk, so also at point of lay 50%. Summers and Leeson (1994) found a delay of pullets coming into production with a lower BW, but differences in production were not found by 28 wk of age. This indicates that, although onset of lay was slightly delayed by feeding MWS, the practical relevance is limited.

FCR during the laying period appeared to be improved when COH compared to CWS was fed during the rearing period (Table 8). The difference between these diets was the inclusion of either 3% fine WS or coarse OH. To the authors’ knowledge, no literature is available on the comparison of the effect on performance of these two fiber sources and their cross-over effect from rearing to laying phase. Guzmán et al (2016) found no effect of adding WS as fiber source to the rearing diet on production performance and feed efficiency during the laying period, but rearing diets were offered as mash diets. Their results are therefore difficult to compare to the results of the present study. Guzmán et al. (2015b) reported that WS addition increased gizzard weight during rearing. In the present study, EPG at 16 wk of age was higher in the COH fed pullets compared to CWS. An improved gizzard function might explain the improved FCR for egg production with the COH fed pullets, but in that case we could expect an improved FCR for the MWS fed pullets during lay as well as EPG was higher for MWS than for COH at 16 wk. Since that was not the case, EPG is probably not the explaining factor and the relation between feed form during rearing and FCR for egg production remains unclear.

The present study showed that feeding a coarse mash during rearing is more efficient for proventriculus + gizzard development than adding coarse OH to a crumbled diet (Table 7). Saldaña et al. (2015a) discussed the hypothesis that a less developed gizzard at onset of lay might affect feed intake and have a negative effect on laying performance. Svihus (2011) questioned whether a small gizzard, often observed in birds fed diets lacking structural components, represents an abnormal situation, and thus may result in suboptimal performance. The present study showed that gizzard development at 16 wk of age was not affecting ADFI and laying performance at later age and supports Svihus’ hypothesis that a smaller gizzard at that age does not seem to represent an abnormal situation per se.

### Ca and P Level

#### Productive Performance

The dietary Ca-P level fed during rearing did not affect growth and production performance of pullets during the rearing and laying period (Tables 4 and 8). These findings are in agreement to Punna and Roland (1999, 2000), who found no effect of dietary non-phytate P (**NPP**) level for egg-type pullets on growth performance during rearing and egg production till 48 wk of age for as low as 0.2% NPP. Their study was performed with a constant dietary Ca level, and differed therefore from the present study, but a negative effect on growth performance, egg production, bone mineral content, and bone mineral density was found at 0.1% dietary NPP, without adding phytase, but not at 0.2% NPP. Jing et al. (2018) found no effect of different dietary NPP levels on growth and FCR and indicated that the lowest NPP level tested (0.2% from 0 to 4 wk; 0.175% from 4 to 8 wk, and 0.15% from 8 to 16 wk) was adequate to support healthy growth and development of egg-type pullets. Previous research from our lab found no negative effects on growth and bone characteristics of low P diets in egg-type pullets, but these diets were only fed from 16 to 27 wk of age (Dijkslag et al., 2019). The low dietary P did have small negative effects on egg weight and egg mass production during the start of lay, when maintaining the low P diets during the laying period, where in the present study adequate dietary P was provided. This indicates a dietary available P requirement of rearing egg-type pullets of approximately 0.15 to 0.2%, but Ca requirement remains unclear.

#### BW Uniformity

BW uniformity was 4.5% lower at 6 wk of age and tended to be lower with 5.6% at 32 wk of age when pullets were fed with the low Ca-P level diets. To the author's knowledge no previous studies have reported the effect of dietary Ca or P or both on BW uniformity in poultry. The effect does not seem to be related to growth performance, bone parameters or egg production and remains therefore unclear.

#### Bone Parameters

Tibia ash content at 11 and 16 wk of age, and keel bone ash content at 11 wk of age were found to be higher with the high dietary Ca-P level, which indicates that the low Ca-P level was slightly below the dietary level for maximum bone mineralization, although tibia breaking strength was not significantly affected (Tables 5 and 6). Several authors have reported bone mineral contents of egg-type pullets fed different dietary P levels and found no effect on bone ash content (Douglas and Harms, 1986; Keshavarz, 2000; Jing et al., 2018; Dijkslag et al., 2019). However Punna and Roland (2000) found a lower bone mineral content and density when fed 0.1% NPP to egg type pullets, without adding phytase, which is less than 50% of the level tested in the present trial. All trials mentioned here were performed with constant dietary Ca levels, which is a different approach compared to the present study. The egg-type pullet Ca requirement remains unclear, but in broiler chickens a Ca to NPP ratio of 2.0:1 was found to be optimal for BW gain and bone development (Gautier et al. 2017).

There was a feed form × Ca-P level interaction on tibia compression at 32 wk of age. At low dietary Ca-P, compression was less when fed crumble (CWS or COH) and numerically more when fed MWS (Table 9). Tibia breaking strength showed a similar direction, but the differences are not significant. This means less energy was needed to break the bone. These findings are in contrast to previous internal research, where no effect of dietary P level on tibia compression was found, but that trial was performed with a constant Ca level (Dijkslag et al., 2019). Thus, Ca and P might have an effect on compression as both elements are required for bone mineralization.

#### Proventriculus + Gizzard Development

Relative EPG was higher when fed low dietary Ca-P at 6 wk of ages, but not at later age (Table 7). Internal research showed a higher relative gizzard weight at 22 wk of age when pullets were fed low dietary P, but the effect interacted with diet particle size, as the difference only appeared when coarsely ground diets were fed (Dijkslag et al., 2019). Birds fed low P diets need to hydrolyze a larger amount of phytate in order to fulfill their P requirement. The pH is an important factor influencing phytate solubility, being more soluble at lower pH (Humer et al., 2015). It can be hypothesized that an increased requirement for hydrochloric acid to solubilize phytate might have stimulated early proventriculus + gizzard development, but the real cause of the effect remains unclear.

#### Laying Period: Cross-Over Effects From Rearing Period

Egg shell quality at 32 wk of age was affected by dietary Ca-P level during rearing. Egg shell breaking strength, egg shell thickness, and SWUSA were improved when low level Ca-P were fed during rearing (Table 10). These findings seem to be in agreement with Miller and Sunde (1975), who reported the lower percentage of poorly shelled eggs, when fed the lowest Ca-P level during rearing. However, previous internal research showed no effect of a lower dietary P level on egg shell breaking strength with the low P level being fed after 16 wk of age (Dijkslag et al., (2019). There is limited data available on the cross-over effect of dietary Ca and P during rearing on egg shell quality at later age. Rousseau et al. (2016) found a significant increase in the mRNA levels of several genes encoding Ca and P transporters in broiler chickens at 36 d of age fed low Ca and P diets from 10 to 21 d. They concluded that chickens are able to adapt to early dietary changes in Ca and P through improvement of digestive efficiency that may persist in a later phase. This mechanism might also work in pullets and may explain the improved egg shell quality at 32 wk when fed low Ca-P during rearing in the present study.

## CONCLUSIONS

From this experiment, we can conclude that feeding crumbles compared to mash increased ADG and ADFI of egg-type pullets during rearing, but this did not clearly affect subsequent egg production performance. There was a clear age effect of feed form, as young pullets showed improved growth performance on crumbles prior to 11 wk of age, although thereafter, birds performed better on mash diets. Replacing finely ground WS by OH as fiber source in the diet did not affect performance during rearing, but some improvement was shown on FCR during lay. Low dietary Ca-P during rearing had no clear effect on pullet development and egg production, but some improvements on egg shell quality were identified at 32 wk of age. This fact deserves further investigation to understand the mode of action behind. Also, low Ca-P during rearing led to lower bone mineralization at 11 and 16 wk of age, although bone breaking strength was unaffected. This indicates that the low Ca-P levels fed during rearing were slightly below the dietary level for maximum bone mineralization, but not for normal BW development.
